# COVID-19: a hypothesis regarding the ventilation-perfusion mismatch

**DOI:** 10.1186/s13054-020-03125-9

**Published:** 2020-07-06

**Authors:** Mario G. Santamarina, Dominique Boisier, Roberto Contreras, Martiniano Baque, Mariano Volpacchio, Ignacio Beddings

**Affiliations:** 1 Radiology Department, Hospital Naval Almirante Nef, Subida Alessandri S/N, Viña Del Mar, Chile; 2 Radiology Department, Hospital Dr. Eduardo Pereira, Enrique Ibsen S/N, Valparaíso, Chile; 3 Intensive Care Unit, Hospital Naval Almirante Nef, Subida Alessandri S/N, Viña Del Mar, Chile; 4 Intensive Care Unit, Hospital San Martín de Quillota, La Concepción 1050, Quillota, Chile; 5 Intensive Care Unit, Hospital IESS Los Ceibos, Av. El Bombero Km 6.5, Guayaquil, Ecuador; 6 Radiology Department, Centro de Diagnóstico Dr. Enrique Rossi, Arenales, 2777 Ciudad de Buenos Aires, Capital Federal Argentina Argentina; 7 Radiology Department, Clínica Bupa Santiago, Av. Departamental 1455, La Florida, Santiago, Región Metropolitana Chile

**Keywords:** COVID-19, Coronavirus, Angiotensin converting enzyme 2, Angiotensin II, Vasoconstriction, Vasoplegia, Ventilation-perfusion ratio

## Manuscript

In December 2019, a novel human coronavirus, SARS-CoV-2, was detected in the city of Wuhan, China, which since then has expanded throughout the world and caused a pandemic coronavirus disease (COVID-19).

SARS-CoV-2 binds to angiotensin-converting enzyme 2 (ACE2) as the functional receptor for cell entry. In contrast to SARS-CoV, SARS-CoV-2 forms more molecular interactions with ACE2, which correlates with data showing a fourfold higher affinity for receptor binding. Subsequently, endocytosis of the viral complex occurs, and surface ACE2 is downregulated. This hampers angiotensin II cleavage, leading to increased circulating angiotensin II and increased angiotensin II receptor activation [1].

ACE2 is a counterregulatory enzyme that degrades angiotensin II to angiotensin-(1-7). Angiotensin-(1-7) stimulates vasodilatation and nitric oxide production and also attenuates the effects of angiotensin II of vasoconstriction, sodium retention, and fibrosis [2]. A study showed that patients with COVID-19 appeared to have elevated levels of plasma angiotensin II, which were correlated with the degree of lung injury and total viral load [3]. SARS-CoV-2 binding to ACE2 may attenuate ACE2 activity, increasing angiotensin II-mediated pulmonary vasoconstriction, as well as inflammatory and oxidative organ damage, ultimately progressing towards acute lung injury and respiratory distress [4]. Decreased activity of ACE2 leads to heightened and relatively unopposed vasoconstriction, pro-coagulation, pro-inflammatory, and pro-oxidant angiotensin II effects [5, 6].

In the alveoli, active replication and release of the virus cause the host cell to undergo pyroptosis, a highly inflammatory form of programmed cell death, releasing damage-associated molecules. These are recognized by epithelial cells, endothelial cells, and alveolar macrophages, triggering the generation of pro-inflammatory cytokines and chemokines, which attract monocytes, macrophages, and T cells to the site of infection, promoting further inflammation and establishing a pro-inflammatory feedback loop. Furthermore, pyroptosis of epithelial and endothelial cells damages the alveolar-capillary barrier, resulting in vascular leakage and alveolar edema [1, 7]. The accumulation of fluid, debris, and inflammatory cells in the damaged lung parenchyma results in the appearance of ground-glass opacities, consolidation, and septal thickening in classic imaging modalities.

A defective immune response may lead to further accumulation of immune cells in the lungs, causing overproduction of pro-inflammatory cytokines with significant damage to the lung structure, leading to a sustained inflammatory response that can result in a cytokine storm, which extends to the rest of the systems causing multi-organ damage [1, 7]. In addition, it has been suggested that patients with SARS-CoV-2 infection suffer a generalized thrombotic microvascular injury, probably mediated by activation of complement pathways and an associated pro-coagulant state. This correlates with the presence of platelet-fibrin thrombi in the small arterial vessels in lung necropsies [8] and is also consistent with very high d-dimer levels found in almost all examined patients. Whether this phenomenon occurs in the absence of macrovascular disease with pulmonary embolism and/or deep venous thrombosis is uncertain, but likely.

Vessel enlargement has been described in the vicinity of areas with ground-glass opacities, which suggests thrombo-inflammatory processes. Subsegmental vascular enlargement (more than 3 mm diameter) in areas of parenchymal lung opacities has been observed in 89% of patients with confirmed COVID-19 pneumonia. Although in situ thrombosis is certainly a possibility, these findings could reflect hyperemia and increased blood flow due to pro-inflammatory factors and vasoplegia induced by SARS-CoV-2 [9].

Advanced imaging techniques can help us visualize perfusion abnormalities that lead to a ventilation/perfusion (V/Q) mismatch in SARS-CoV-2 infection, both in the abnormal parenchyma and in the apparently normal parenchyma. A recent publication described perfusion abnormalities in COVID-19 infection using dual-energy computed tomography in relation to areas of injured parenchyma [10]. Subtraction computed tomography (SCT) is another imaging modality that uses software-based motion correction between sets of unenhanced and contrast-enhanced CT scan images for obtaining the iodine distribution in the pulmonary parenchyma [11].

We apply SCT routinely in COVID-19 patients who undergo CT angiography, obtaining iodine distribution maps in the lung parenchyma. Arithmetic subtraction of the precontrast image from the contrast-enhanced image is performed using 100-kV single-energy CT acquisitions, with motion correction using the SURE Subtraction Lung algorithm (version 7; Canon Medical Systems, Otawara, Japan).

**Fig. 1:**
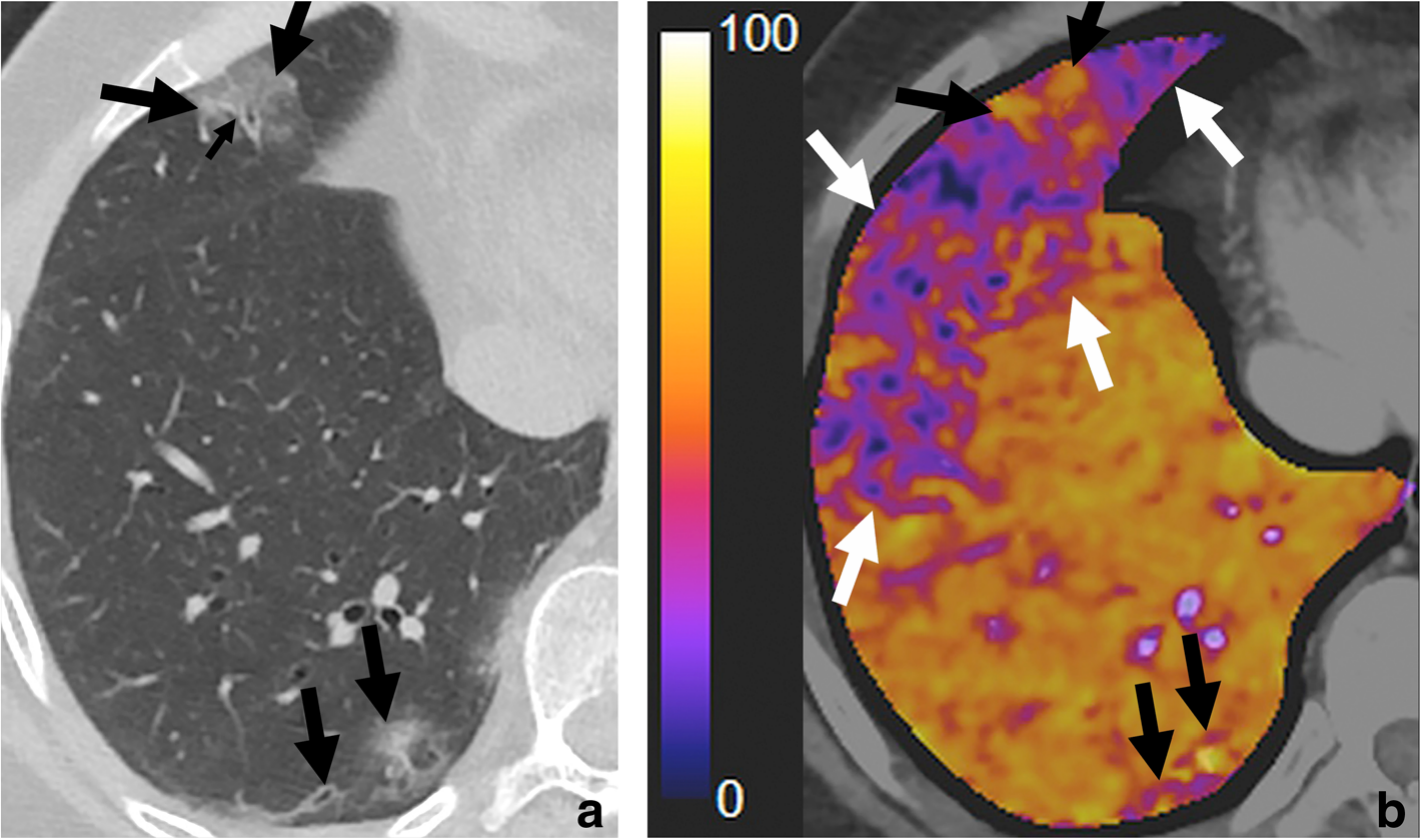
**a**, **b** Slight hypoperfusion in the well-aerated lung, hyperemia, and small zones of hypoperfusion in the areas of injured lung. Fifty-nine-year-old male patient, RT-PCR-confirmed COVID-19, 11 days since symptom onset, without hypoxemia, (PaO_2_/FiO_2_) 538, d-dimer 340 ng/mL. There are isolated foci of ground-glass opacities associated with septal thickening, with a predominantly subpleural distribution, which correlate with areas of hyperemia (middle lobe) and small zones of hypoperfusion (lower right lobe) in subtraction CT iodine maps (large black arrows). There is an evident area of hypoperfusion in the middle lobe and lower right lobe (white arrows) that correlates with the apparently normal lung parenchyma in conventional chest CT images. The conventional CT image also shows pulmonary arterial vascular dilatation in the periphery of the ground-glass opacity in the middle lobe (small black arrow). These slight perfusion abnormalities do not impact the PaFi ratio. The ground-glass opacity in the lower right lobe shows slight peripheral hypoperfusion, probably due to compensatory vasoconstriction, an expected regulatory mechanism when vasoplegia is not fully established

**Fig. 2:**
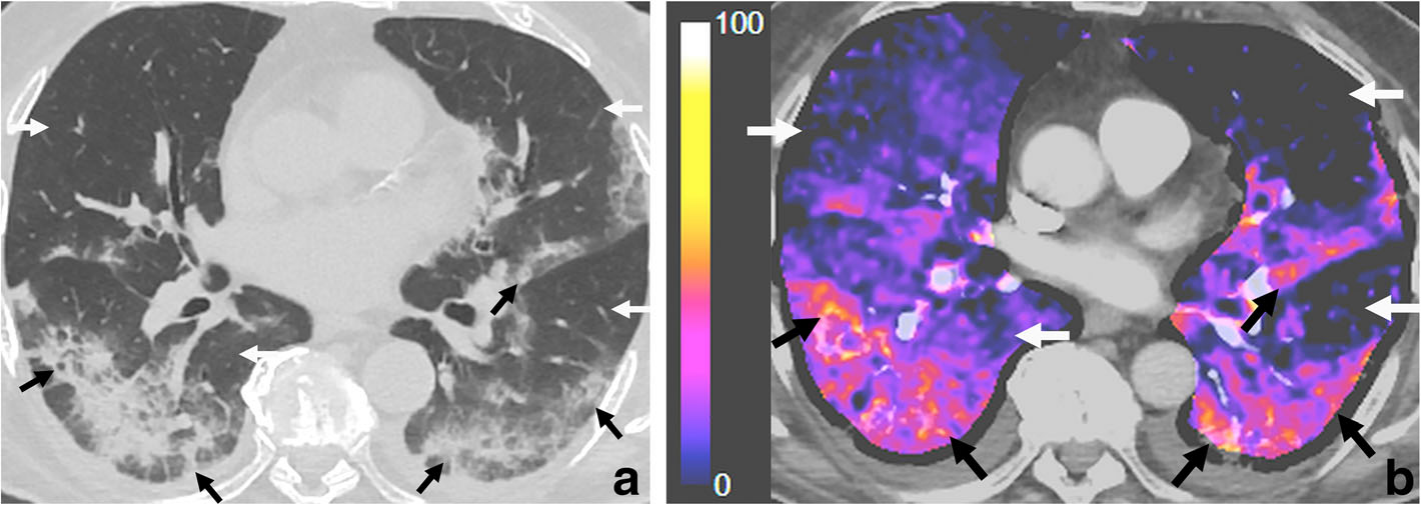
**a**, **b** Prominent hypoperfusion in the well-aerated lung and hyperperfusion in areas of injured lung. Seventy-eight-year-old male patient, RT-PCR-confirmed COVID-19, 10 days since symptom onset, with hypoxemia, (PaO_2_/FiO_2_) 206, d-dimer 1600 ng/mL progressively increasing. There are extensive foci of consolidation and ground-glass opacities, associated with septal thickening, with a predominantly posterior and subpleural bilateral distribution, which correlate with the areas of hyperemia and iodine pooling in subtraction CT iodine maps (black arrows). There are areas of markedly decreased perfusion in both lungs, which correlate with the apparently healthy lung parenchyma in conventional chest CT images (white arrows). Bilateral pleural effusion. This could be explained by an increased blockage of ACE2 receptors in the lung endothelium, leading to increased local levels of angiotensin II, which leads to vasoconstriction and ventilation/perfusion mismatch. This patient was managed with invasive mechanical ventilation, with highly compliant lung parenchyma, in accordance with the type 1 or L phenotype described by Gattinoni et al.

Gattinoni et al. describe COVID-19 pneumonia as a specific disease characterized by severe hypoxemia, often associated with near-normal respiratory system compliance (type 1 or L), different from classical ARDS [12]. In this subgroup of patients, we have found abnormal hyperperfusion in areas of lung opacities in patients with hypoxemia in a similar fashion to what has been described recently [10]. However, we have also found abnormally decreased iodine distribution in areas of the apparently normal lung parenchyma, which worsens in more severe cases (Figs. 1 and 2).

We believe that a severe V/Q mismatch underlies the pathophysiology of moderate to severe COVID-19 cases, in which downregulation of ACE2 secondary to viral endocytosis plays a key role. There is low V/Q ratio in areas of injured lung parenchyma with ground-glass opacities or consolidation, secondary to loss of compensatory hypoxic pulmonary vasoconstriction (vasoplegia) and increased blood flow, which result in high perfusion to the areas of non-aerated lung, but there is also high V/Q ratio in areas of apparently healthy lung secondary to prominent vasoconstriction. We hypothesize that hypoperfusion of apparently healthy areas could be a consequence of vasoconstriction due to accumulation of angiotensin II, caused by decreased availability of ACE2, and that these changes in vascular resistance lead to a shunt or steal of vascular flow towards areas of non-aerated hyperperfused lung in moderate to severe COVID-19 cases.

The improved oxygenation in prone position that has been described in patients with COVID-19 could be explained mainly through vascular redistribution towards the areas of apparently healthy lung with a high V/Q ratio, rather than alveolar recruitment.

These findings may support the early use of pulmonary vasodilators, such as inhaled nitric oxide and prostacyclin, to improve the ventilation/perfusion mismatch, and provide initial insight into the complex viral pathophysiology of hypoxemia and perfusion abnormalities in COVID-19.

Further studies are needed to investigate ventilation-perfusion abnormalities and whether these could be explained by the local increase in angiotensin II.

## Data Availability

Data sharing is not applicable to this article as no datasets were generated or analyzed during the current study.

## Abbreviations

ACE2: Angiotensin-converting enzyme 2
V/Q: Ventilation/perfusion
SCT: Subtraction computed tomography
